# Denosumab Re-Challenge and Long-Term Efficacy for Aneurysmal Bone Cyst of the Spine: Enhanced Treatment Algorithm

**DOI:** 10.3390/jcm13154522

**Published:** 2024-08-02

**Authors:** Gisberto Evangelisti, Franziska C. S. Altorfer, Luigi Falzetti, Emanuela Palmerini, Cristiana Griffoni, Riccardo Ghermandi, Stefano Boriani, Annalisa Monetta, Marilena Cesari, Toni Ibrahim, Alessandro Gasbarrini

**Affiliations:** 1 Department of Spine Surgery, IRCCS Istituto Ortopedico Rizzoli, 40136 Bologna, Italy; gisberto.evangelisti@ior.it (G.E.); luigi.falzetti@ior.it (L.F.); riccardo.ghermandi@ior.it (R.G.); annalisa.monetta@ior.it (A.M.); alessandro.gasbarrini@ior.it (A.G.); 2 University Spine Center Zürich, Balgrist University Hospital, University of Zürich, 8006 Zürich, Switzerland; altorferf@hss.edu; 3 Osteoncology, Bone and Soft Tissue Sarcomas and Innovative Therapies Unit, IRCCS Istituto Ortopedico Rizzoli, 40136 Bologna, Italy; marilena.cesari@ior.it (M.C.); toni.ibrahim@ior.it (T.I.); 4 Post Graduate Program of Orthopedics at IRCCS Istituto Ortopedico Rizzoli, University of Bologna, 40123 Bologna, Italy; sb@stefanoboriani.eu; 5 Department of Biomedical and Neuromotor Sciences, University of Bologna, 40123 Bologna, Italy

**Keywords:** aneurysmal bone cysts, spine, denosumab, recurrence, re-challenge, treatment algorithm

## Abstract

**Background/Objective:** Surgical treatment of aneurysmal bone cysts (ABCs) can be challenging, especially in the spine. Non-surgical treatments such as with denosumab have shown promising results in different osteolytic pathologies. This retrospective observational study aimed to evaluate the long-term clinical and radiologic response of patients with ABCs of the mobile spine treated with denosumab and propose an updated treatment algorithm. **Methods:** Six patients with relapsed and symptomatic ABCs of the mobile spine were treated with denosumab (120 mg subcutaneously on days 1, 8, 15, 29, and every 4 weeks thereafter) between 2012 and 2023. Disease assessments were conducted using CT and MRI at 3, 6, 9, and 12 months post-treatment. Clinical data, including pain levels, symptoms, and adverse events, were documented from patients’ charts. **Results:** Patients underwent an initial phase of treatment with denosumab, receiving a mean of 22 administrations (range 13–42) over a median follow-up period of 41 months (range 15–98 months). Clinical improvement was observed in all patients after 4 weeks of treatment, and all patients demonstrated a radiological response after 12–24 weeks on denosumab. Three patients were progression-free after discontinuing denosumab following 13, 15, and 42 administrations, respectively. At the last follow-up, after 38, 43, and 98 months, these patients remained stable without relapse of the disease. Three patients had a relapse of disease after denosumab; two of them underwent denosumab re-challenge, while one patient received one mesenchymal stem cells (MSCs) injection. All patients showed clinical and radiological improvement and were resulted to be disease-free at the last follow-up. **Conclusions:** This study demonstrates the long-term efficacy and safety of denosumab in treating ABCs of the mobile spine, as well as the potential of re-challenge in managing recurrence. A treatment algorithm is proposed, positioning denosumab as a viable therapeutic option after other local treatments. Careful patient selection, monitoring, and further research are necessary to optimize denosumab use for ABCs.

## 1. Introduction

Aneurysmal bone cysts (ABCs) are rare conditions, and present a significant challenge, particularly when localized in the spine [1,2]. ABCs are benign tumors with potential locally aggressive behavior characterized by loculated blood-filled cystic areas. They represent 1.4% of primary bone tumors (15% of spinal bone tumors), with a prevalence of 0.32/100,000 per year in the young population and 0.14 per 100,000 in the general population (192 patients/year) in the USA [3]. Accounting for 15% of spinal bone tumors, these lesions often lead to debilitating pain and functional impairment, necessitating prompt and effective intervention. In spinal localization, neurologic deficits may be caused by compression of nerve roots. The treatment of spinal ABCs presents unique challenges due to their location and proximity to critical neurological structures. Traditional surgical approaches, while effective, can be associated with significant morbidity, including potential neurological deficits, spinal instability, and prolonged recovery periods. These factors underscore the need for less invasive, yet equally effective treatment modalities. Commonly accepted non-surgical treatments such as embolization and injection of autologous stem cells or sclerosant agents have been the mainstay for managing spinal ABCs [4,5]. In 2017, Terzi et al. reported an algorithm for treating spinal localization of ABCs, which is still used nowadays; however, due to the complex nature of spinal lesions and the potential for recurrence, exploring targeted and innovative therapies becomes imperative [6]. An ABC contains osteoclast-like multinucleated giant cells (MNGCs) and fibroblast-like cells, similar to giant-cell tumor of bone (GCTB) [7,8]. Since osteoclasts are the only cells responsible for bone resorption, the MNGCs within GCTB and ABCs appear responsible for these tumors’ osteolytic nature [8,9,10].

Like GCTB, the receptor activator of the nuclear factor kappa-B (RANK) ligand is highly expressed in the stroma of ABCs. It dictates the activation of MNGCs, binding to RANK on the surface of monocyte and macrophage lineage precursors. The RANK signaling pathway has an essential role in tumor progression [8,9,10,11].

Denosumab, a RANK ligand inhibitor, is approved for the treatment of osteoporosis and prevention of skeletal-related events in patients with multiple myeloma and in patients with bone metastases from solid tumors [12]. Denosumab is a fully human monoclonal antibody that inhibits osteoclast maturation, activation, and function by binding to the receptor activator of the nuclear factor kappa B ligand, with the final result being a reduced rate of bone resorption [13]. It has successfully managed GCTB and is approved for GCTB treatment [14,15,16]. Further, denosumab has proven to be an off-label use sufficient in treating spinal ABCs, resulting in pain relief and neurological improvement in short-term follow-up [17,18,19,20]. However, data for long-term follow-up of denosumab treatment of ABCs of the spine are lacking. The emergence of denosumab as a potential therapy for spinal ABCs represents a paradigm shift in management strategies. By targeting the RANK/RANKL pathway, denosumab offers a molecular approach to treatment that could potentially reduce tumor size, alleviate symptoms, and stabilize lesions without the immediate risks associated with invasive procedures. Furthermore, in cases where surgery is eventually required, preoperative denosumab treatment may facilitate less extensive and safer surgical interventions by reducing tumor vascularity and promoting ossification [17]. However, the long-term efficacy, optimal treatment duration, and potential side effects of denosumab in the context of spinal ABCs remain areas of active research and clinical interest.

The purpose of this study is to assess the long-term clinical and radiological outcomes of patients with ABCs of the spine who were treated with denosumab, a medication used when surgical intervention was either not possible or would have resulted in significant complications and morbidity.

## 2. Materials and Methods

The off-label use of denosumab was approved upon request by the regional committee for each patient and informed consents were obtained. Data were collected retrospectively as part of a single center observational study (registry) approved by the local ethics committee in December 2016 (protocol number 0022814). This study included patients with ABCs of the mobile spine above the sacrum, treated between October 2012 and July 2023.

In all cases, a CT-guided needle biopsy was performed, and histological diagnosis was confirmed by expert pathologists.

**Figure 1:**
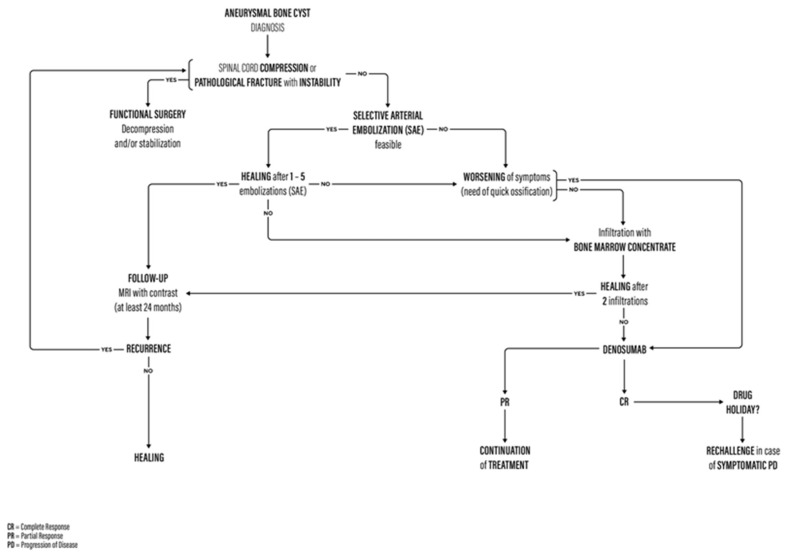
Flowchart for the management of ABCs of the spine.

The inclusion criteria were the following: the absence of pathological fractures, spine instability, symptomatic spinal cord compression, and the failure of previous treatments, which encouraged the clinicians to start a therapy with denosumab (according to the flowchart in Figure 1). The minimal follow-up was 12 months following the start of denosumab treatment. Exclusion criteria were the following: lost to follow-up, as well as allergies to denosumab.

*Denosumab treatment*

After the failure of previous treatments (see treatment flowchart in Figure 1) and after a multidisciplinary evaluation by an oncologist, radiologist, spine surgeon, and radiotherapist, treatment with denosumab was initiated. Denosumab was administered in 4 weekly cycles with subcutaneous administration of 120 mg on days 1, 8, 15, and 29, and afterward changed to the same dose every 4 weeks. To prevent hypocalcemia, patients received daily supplementations of 500 mg of calcium and 400 IU of vitamin D, as per GCTB trials [15,20].

*Outcomes assessment*

For this study, a quantitative evaluation of the gadolinium changes pre- and post-treatment was not conducted. Instead, a qualitative assessment of these changes was performed. This evaluation was carried out by a multidisciplinary team consisting of radiologists, spine surgeons, and oncologists. Disease evolution was assessed by computed tomography (CT) scans and/or magnetic resonance imaging (MRI) at 3, 6, 9, and 12 months post-treatment, and then every 6 or 12 months, following the staging proposed by Boriani et al. for the evaluation of the radiological response of spinal GCTB to denosumab therapy [2].

Healing signs, including peripheral sclerotic bone rim formation, reduction in ABC mass, disappearance of the double content image, bone formation inside the ABC mass, and pain remission, determined the treatment’s success. Once these criteria were met, patients underwent further monitoring with MRI every 6 months for an additional 2 years. All imaging data were centrally reviewed for the study without blinding to clinical information.

Detailed records of clinical data, encompassing pain evaluation, ability to perform daily activities, and denosumab-related adverse events were documented from patients’ charts.

## 3. Results

*Patients*

Six patients (five male and one female) treated with denosumab for spinal ABCs were retrospectively identified. As reported in Table 1, no patient presented comorbidities or smoking habit. The localization of the ABC was the cervical spine in three cases, the lumbar spine in two cases, and the thoracic spine in one case. All patients were previously treated for spinal ABCs at our institute with selective arterial embolization (SAE) and/or MSCs injection (Table 1).

Two patients had previously undergone curettage and stabilization in another hospital and had been referred because of a local recurrence. We have included these “non-intact” cases in the present study because the clinical onset of local recurrence and the therapeutic planning were comparable to untreated ones.

Due to the failure of previous treatments (SAE and/or MSCs injection) and the local recurrence of ABCs, the six patients of this cohort were treated with denosumab, as reported in Table 2. The median age at the start of denosumab treatment was 22.5 years (range 16–42 years).

*Treatment and Outcome*

As reported in Table 2, the patients underwent an initial phase of treatment with denosumab, receiving a mean of 22 administrations (range 13–42) over a median follow-up period of 41 months (range 15–98 months). No significant adverse events were collected. Clinical improvement, in particular pain relief, was observed in all patients after 4 weeks of treatment, and all patients demonstrated a radiological response after 12–24 weeks on denosumab. CT scans showed bone formation in all patients, and a decrease in MRI gadolinium contrast media was seen in all six patients. Sustained tumor control was demonstrated in all patients.

Three patients were progression-free after discontinuing denosumab following 13, 15, and 42 administrations, respectively. No progression was documented after a median of 12 months (range 6–12 months) following denosumab interruption, and no surgeries were performed. At the last follow-up, after 38, 43, and 98 months, these patients remained stable without relapse of the disease (Table 2). No pain and no limitations in daily activities were recorded at last follow-up. Denosumab treatment was stopped in these three patients due to the radiological and clinical improvement in their disease. In the absence of specific guidelines for discontinuing denosumab in cases of ABCs of the spine, the decision to stop treatment was made on a case-by-case basis by a multidisciplinary team.

This team, consisting of oncologists, spine surgeons, and radiologists, evaluated each patient’s individual response to treatment. The marked improvement in both radiological findings and clinical symptoms was the primary factor in deciding to cease treatment at these specific time points.

It is important to note that the variation in treatment duration (13, 15, and 42 months) reflects the individualized nature of each patient’s response and the careful monitoring process. The decision to stop treatment was not based on a predetermined timeframe, but rather on each patient’s unique disease progression and response to therapy.

These patients continue to be closely monitored for any signs of disease recurrence or progression, with the understanding that treatment could be reinitiated if necessary. This approach underscores the need for personalized treatment strategies in managing spinal metastases with denosumab.

*Relapsed Cases*

Three patients had a relapse of disease after denosumab treatment (Table 2). Two patients with local recurrence underwent denosumab re-challenge, which means restarting denosumab after discontinuing it, while one patient received one mesenchymal stem cells (MSCs) injection.

**Figure 2:**
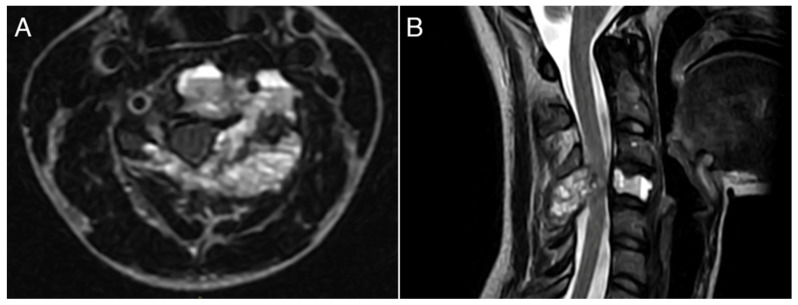
A 26-years-old woman presented with neck pain and no neurological problems. MRI pattern was pathognomonic for ABC. (**A**): axial view; (**B**): sagittal view.

**Figure 3:**
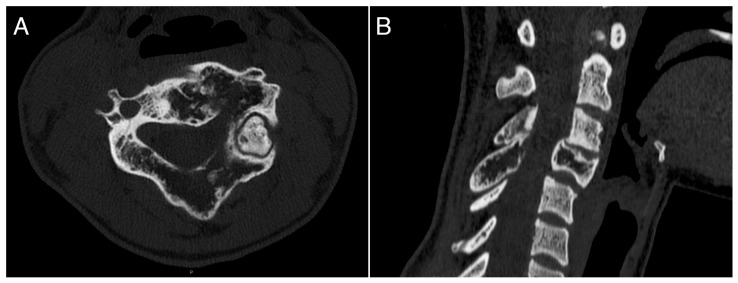
CT scan performed at seven months of treatment with denosumab, showing significant ossification of the lesion. (**A**): axial view; (**B**): sagittal view.

**Figure 4:**
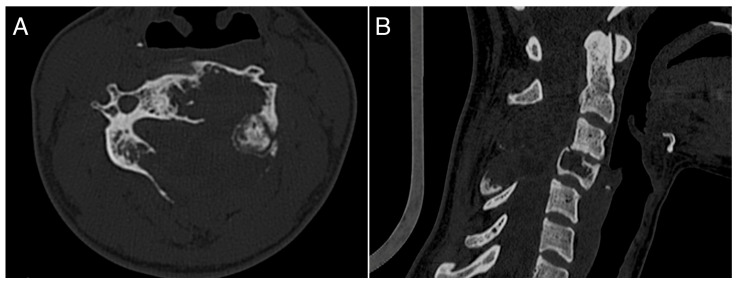
CT scan showing a local recurrence occurring ten months after stopping denosumab treatment. (**A**): axial view; (**B**): sagittal view.

**Figure 5:**
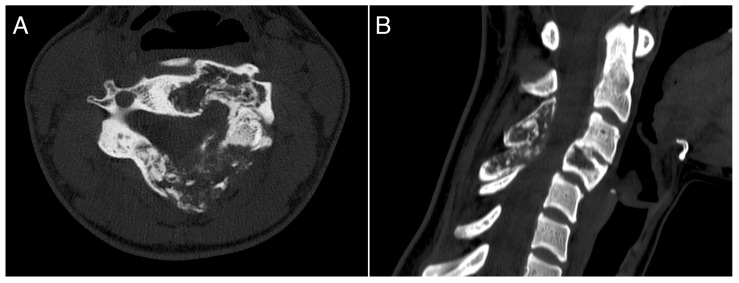
CT scan performed at last follow-up 24 months after denosumab re-challenge. (**A**): axial view; (**B**): sagittal view.

The three cases are described in detail:

A 26-year-old female with a massively lytic lesion in C4 showed consistent ossification after seven months of treatment (Figure 2 and Figure 3). She chose to discontinue denosumab after 20 administrations due to being asymptomatic and being at her fertile age. However, ten months after stopping the medication, she experienced severe neck and upper left pain, leading her to seek urgent medical attention at the emergency department of our clinic. CT and MRI showed a local recurrence of the ABC in C4 (Figure 4 and Figure 5). She then received a “re-challenge” treatment with denosumab according to the above-mentioned protocol, and the treatment is still ongoing. At the last follow-up, 20 months after being on denosumab with a monthly schedule, the patient was asymptomatic, and the last CT showed good ossification of C4 (43 months follow-up from the first treatment).

A 42-year-old male presented a lytic lesion in C7, which exhibited complete ossification after 27 administrations over 5 years of denosumab therapy. Encouraged by the extended period of well-being, the patient requested to withdraw from the protocol. However, fifteen months after discontinuation, a follow-up MRI revealed a local recurrence of the ABC in C7. Subsequently, the patient underwent denosumab “re-challenge”, and the treatment is still ongoing. At the last follow-up, 24 months after initiating denosumab with a monthly schedule, the patient was asymptomatic, and the CT scan showed complete ossification (103 months follow-up from the first treatment).

A 25-year-old male patient with a lytic lesion in T10 exhibited complete ossification of the lesion after 18 administrations over a 3-year period of denosumab therapy. Encouraged by the prolonged well-being and the complete ossification, the patient opted to withdraw from the protocol. However, twenty months after discontinuation, a follow-up MRI revealed an asymptomatic area of local recurrence of the ABC in T10. Due to the localized recurrence, easily accessible with a CT-guided needle, and following a multidisciplinary evaluation, the patient underwent an injection of autologous stem cell concentrate. At the last follow-up, conducted 12 months after the procedure, the patient remained asymptomatic and showed radiological improvement.

No significant adverse events requiring discontinuation of denosumab treatment were detected in the long-term period.

## 4. Discussion

This study provides insights into the use of denosumab as a treatment option for ABCs of the mobile spine. Its results indicate that denosumab led to significant clinical improvement with notable radiological responses in a relatively short timeframe.

Three out of six patients treated with denosumab had local relapse 10 to 20 months after drug discontinuation. In two of these three cases, subsequent denosumab re-challenge was administered, again with radiological response and relief from symptoms. The remaining three patients showed no disease progression after denosumab discontinuation, with confirmed stable conditions at a long-term follow-up. Denosumab treatment was stopped after 13, 15, and 42 months in these three patients due to the radiological and clinical improvement in their disease. In the absence of specific guidelines for discontinuing denosumab in cases of spinal ABCs, the decision to stop treatment was made on a case-by-case basis by a multidisciplinary team. The marked improvement in both radiological findings and clinical symptoms was the primary factor in deciding to cease treatment at these specific time points. These patients continued to be closely monitored for any signs of disease recurrence or progression, with the understanding that treatment could be reinitiated if necessary.

These findings have several important implications for clinical practice in the management of spinal ABCs. Firstly, the potential for lasting effects of denosumab treatment in selected cases beyond the active treatment phase, given also the relatively rapid clinical improvement and radiological responses observed, suggests that it could be considered as a primary treatment option for patients with spinal ABCs not suitable for embolization or surgery. This is particularly relevant for cases where surgical intervention poses substantial risks of neurological deficits or major complications.

Secondly, the varying responses to denosumab treatment observed in our study highlight the need for careful patient selection and monitoring. Patients with lesions considered not suitable for embolization, surgery, or injection with local agents may be prime candidates for denosumab therapy. However, the potential for relapse after discontinuation underscores the importance of developing individualized treatment plans with close follow-up. For patients who remained on denosumab treatment, our study’s follow-up showed long-term disease control without severe toxicity.

Furthermore, our experience with denosumab re-challenge suggests that this could be a viable strategy for patients who experience relapse after initial response and treatment discontinuation. This approach could be particularly valuable in managing long-term disease control while minimizing cumulative drug exposure and associated risks.

Lastly, the possibility of combining denosumab with other treatments, as demonstrated in one of our cases, opens up new avenues for personalized treatment strategies. Clinicians should consider the potential synergistic effects of combining denosumab with other modalities such as autologous stem cell injections or minimally invasive surgical techniques.

One patient in the presented cohort received an autologous stem cell injection after detecting an asymptomatic local recurrence. This case shows that treatment with denosumab might likely be combined with other treatments.

The choice between treatments depends on the clinical scenario: If the lesion is asymptomatic and does not require rapid ossification, autologous stem cells are the preferred option. However, if the patient is symptomatic and the lytic aspect of the lesion poses a risk of fracture, denosumab may be a better option due to the rapid response, both clinically and radiologically, observed in all patients (decreased pain and paresthesia, bone formation visible on CT scans, and reduced gadolinium contrast uptake on MRI).

Previous reports have described individual cases where denosumab was utilized to manage ABCs [9,21,22]. Pelle et al. documented a case involving a 5-year-old boy with a sacral ABC, reporting improvements in pain and neurologic symptoms within weeks of denosumab treatment, leading to a reduction in tumor volume observed on MRI [9]. Similarly, Pauli et al. reported successful management of a 21-year-old woman with local recurrence of a proximal forearm ABC, wherein denosumab treatment facilitated surgery by better delimitating the tumor with a bony rim after 5 months [21]. Additionally, Ghermandi et al. highlighted denosumab’s efficacy in two cases with sacral ABCs, demonstrating lesion healing, regression of neurologic deficits, and pain relief [22]. The authors concluded that denosumab therapy was clinically and radiologically effective and could surface as a potential substitute therapy in patients with SAE-resistant ABCs. Palmerini et al. and Kurucu et al. reported the largest case series, nine and eight patients, respectively, focusing on the use of denosumab specifically on ABCs in the appendicular and axial skeleton, including cases of spinal localization [7,18]. In addition to these data, our study represents the largest case series focusing on spine patients only, offering long-term follow-up data and, to our knowledge, it is the first report of denosumab re-challenge after the discontinuation of treatment of the ABCs. This is consistent with data from one study of inoperable or locally advanced giant cell tumor of bone, where 13 patients underwent successful denosumab re-challenge [23].

There are unanswered questions on the systemic treatment of ABCs with denosumab, such as the duration of the treatment after symptomatic and radiologic response.

Shorter (i.e., 6 months) durations of treatment, drug holidays, and re-challenge in selected progressing patients might reduce denosumab’s long-term side effects such as osteonecrosis of the jaw, which is dose-dependent [15].

We propose an updated version of the treatment algorithm previously presented by Terzi et al. in which denosumab is positioned after the other already established effective treatment options for ABCs of the spine (see Figure 1) [6].

The limitations of the present study are the relatively small sample size and the absence of a control group for comparative analysis. Further research, including larger prospective studies and comparative analyses, is warranted to validate these findings and address the limitations observed in this study. Establishing a specific timeline for the therapy, including both a minimum and a maximum duration, will be of paramount importance to ensure clarity and effective management. The flow-chart presented here summarizes the authors’ experience and the most recent literature findings, and it is not intended to provide recommendations.

## 5. Conclusions

In conclusion, this study provides valuable evidence supporting the long-term efficacy and the safety of denosumab treatment and re-challenge for ABCs of the mobile spine. The observed clinical and radiological responses, along with the documented sustained tumor control, emphasize denosumab’s potential as a viable therapeutic option for patients with ABCs. However, careful patient selection, continuous monitoring, and further research are imperative to optimize denosumab’s use, ensuring the best possible outcomes for patients affected by this condition.

## Figures and Tables

**Table 1:** Demographics and clinical data of patients before denosumab treatment.

Patient ID	Age at Diagnosis	Comorbidities	Smoking	Date of ABC Diagnosis	Localization of ABC	Previous Treatments *
1	15	NO	NO	January 2013	L5-S1	5 SAE
2	18	NO	NO	September 2016	L3-L4	5 SAE 2 MSCs injections
3	19	NO	NO	November 2019	C6-C7	debulking and stabilization 1 SAE
4	40	NO	NO	May 2012	C7	debulking and stabilization 2 SAE
5	24	NO	NO	January 2015	T10	3 SAE 2 MSCs injections
6	26	NO	NO	November 2019	C4	1 MSCs injection

* MSC = Mesenchymal stem cells; SAE = Selective arterial embolization.

**Table 2:** Treatment of spine ABC with denosumab.

	Age at Start of Therapy	Site of Tumor	Denosumab * (Number of Administrations)	Radiological Response (Y/N)	Progression-Free Survival (Months)	Recurrence after Suspension (Y/N)	MSCs Administration (Y/N)	Denosumab Re-Challenge (Y/N)	Denosumab Re-Challenge (Number of Administrations)	Radiological Response (Y/N) after Re-challenge/MSCs Injection	Last Follow-up (Months)
	16	L5-S1	42	Y	98	N	N	N	NA	NA	98
	19	L3-L4	13	Y	43	N	N	N	NA	NA	43
	20	C6-C7	15	Y	38	N	N	N	NA	NA	38
	42	C7	27	Y	70	Y	N	Y	24 (ongoing)	Y	103
	25	T10	15	Y	39	Y	1 MSCs injection	N	NA	Y	56
	26	C4	20	Y	15	Y	N	Y	20 (ongoing)	Y	43
mean			22								
median	22.5				41						

* From denosumab 1st administration date to progression or most recent follow-up visit. MSCs = Mesenchymal stem cells; NA = Not applicable.

## Data Availability

Data supporting reported results can be found by asking the corresponding authors.
